# Every picture tells a story: Content analysis of medical school website and prospectus images in the United Kingdom

**DOI:** 10.1007/s40037-019-00528-5

**Published:** 2019-07-25

**Authors:** Jack Macarthur, Mike Eaton, Karen Mattick

**Affiliations:** 1 College of Medicine & Health, University of Exeter, Exeter, UK; 2 Centre for Research in Professional Learning, University of Exeter, Exeter, UK

**Keywords:** Medical education, Images, Prospectuses, Websites

## Abstract

**Introduction:**

The decision-making process for students as to which medical schools to apply to is open to many factors and influences. Research has identified several factors which influence prospective students’ choice of medical school and career. There is also evidence that websites and prospectuses may be creating potential barriers to widening access.

**Methods:**

The websites and prospectuses of 33 medical schools in the United Kingdom were searched for relevant images. These images and the people in them were subjected to inclusion/exclusion criteria. Data about the images and people were recorded so that a content analysis could be performed. The relative proportions were compared with pre-existing data relating to the medical profession and society.

**Results:**

From 33 medical schools, 650 images were included, with 1,817 people depicted. The largest group for the assumed roles was ‘student’, as expected, with 1,423 people (78%). For the overall theme of the image, community placement themed images made up only 2% of images (14) and hospital placement themed images made up 24% (154). Chi-squared goodness of fit showed statistically significant results for most groups of data when comparing ethnicity, the overall theme of the image and assumed specialty group, but not when comparing gender.

**Discussion:**

In conclusion, for gender, medical schools are accurately reflecting national data. However, for ethnicity medical schools fail to accurately represent national data, leading to incorrect signalling about the ethnic makeup of their students. Additionally, medical schools are signalling to students a strong preference for hospital-based settings, despite a strong national drive to recruit more general practitioners.

## What this paper adds

Choosing the ‘right’ students to admit to medical school is critically important. Research has shown that medical schools can influence why people apply to medicine and the specialty they chose. Images are one of the ways that medical schools convey information about themselves and the medical profession to students. This paper adds value to the existing literature by evaluating the images chosen by medical schools for their websites and considering how these might inadvertently affect the choices made by prospective students.

## Introduction

Ensuring the right number and ‘type’ of medical students enter undergraduate training is an important and challenging task. In the United Kingdom (UK) the number of medical students is carefully governed by the Department of Health. In August 2017, the Department of Health announced an increase in the number of places for medical students by 1,500 [1], an increase of 25% from the previous 6,000 places [2]. The allocation of these additional places was determined by several different elements [3] but preference was given to medical schools that showed their recruitment strategy created a future workforce reflecting the population it served [1]. Furthermore, curricula with a greater proportion of general practice placements were favoured since greater recruitment to this specialty is needed and longitudinal placements within general practice have been reported to positively influence medical students’ choice of general practice as their future career [4]. However, it remains to be seen whether this strategy will prove successful in creating a more diverse pool of graduates who are more likely to choose general practice.

To achieve a more diverse workforce in the right specialty areas, we need to understand more about how applicants to medical school make their decisions and how they choose a specialty. There is little large-scale research to date exploring how potential applicants choose a medical school. Foster identified five areas influencing their choice [5]: curriculum factors, the reputation of the school, personal contact, location, and facilities. Similarly, Brown stated three factors influenced prospective students’ choice: academic factors, location factors, and ‘intangibles’ [6]. The first point of contact was usually the medical school website or prospectus, and both studies highlight the importance of an impression formed about a place, for example, based on images, anecdotes, and individual experiences, rather than the more logical-rational decision making one might have anticipated. This is consistent with research done by Cleland et al. [7] which also showed medical schools influenced medical students’ future career choices.

A study by McHarg et al. [8] showed how important having a supportive figure is in applying to medical school, with several students reporting that without the support of someone else they would not have applied. As such it is important that the images used do not create barriers by undermining crucial support.

A study by Chowdhury et al. [9] reviewed images used in prospectuses by medical schools to determine the frequency different learning environments were shown. They found that 46% of prospectuses had images of students in laboratories, 41% had images of students in clinical skills settings, 34% of prospectuses had images of students in a hospital setting and only 6% had images of students in community settings. Alexander et al. [10] showed that websites may be creating a barrier to widening access to medicine through the language that they use. As such it is important to see if the images used on websites are also creating potential barriers as well.

Visual cues are increasingly known to be important influencers of choice in a range of fields [11, 12]. This makes sense considering theories such as signalling theory [13], image theory [14, 15], and social identity theory [16]. Signalling theory states that one party communicates information about itself to another party via signals. The receiver uses the signals to determine the credibility and suitability of the sender. Image theory deals with how objects are assessed to determine the compatibility with the individual’s values and ethics. Compatibility is primarily based upon any violations of compatibility; violations are not compensated for by positive attributes. Social identity theory states people align themselves with a group whom they consider to have similar characteristics to themselves.

It is likely that images on websites and in prospectuses are important influencers of medical school and specialty choice, and as such important in shaping the future workforce. However, we do not know whether these images are aligned with national recruitment priorities and it is possible that website images are undermining efforts to widen access to medicine.

This article aims to answer the research question: to what extent do the images of people used within UK medical school prospectuses and websites reflect current demographic data for medical students and healthcare professionals?

## Methodology

### Study design

This research involves a content analysis of the photographic images embedded within medical school websites and prospectuses. Haggarty defines content analysis as ‘a research method which allows the qualitative data collected in research to be analyzed systematically and reliably so that generalizations can be made from them in relation to the categories of interest to the researcher’ [17].

### Medical schools

The UK Medical School Council website was used to compile a list of UK medical schools [18]. To be included, medical schools had to offer an accredited medical degree with either undergraduate or postgraduate entry and be actively recruiting students. The 33 medical schools were each assigned a number using a random number generator, to protect their identities. For each medical school, the website and prospectus were searched to locate images suitable for this research.

### Image bank

For image collection from websites, only webpages related to the medical course and from the medical school home page were included, all other university or medical school webpages were excluded. For images from prospectuses, the pages included were the title page, any pages up to and including the contents, and pages specifically relating to the medical degree; all other pages were excluded.

The inclusion criteria for images taken from the included webpages/prospectus pages were: the image includes at least one person, and the image is related to the medical degree program. Images were excluded from collection if they were from news articles; about the student experience not related to the medical course or contained more than 15 people.

### Data extraction

Data from the images were extracted and entered into a Microsoft Excel database. For each person in each image, the following information was recorded: assumed role (e.g. student, teacher, doctor, patient, nurse, other); assumed sex; assumed ethnicity category; and assumed age category. For each image, the following information was recorded: the total number of people; total number for each assumed role; and overall theme (academic, community placement, hospital placement, and other). If two or more data points could not be recorded about an individual, then that individual was excluded. Where the assumed role of a person in an image was a doctor, they were further categorized as to their assumed specialty group (community-based or hospital-based). In the database, the UK country (England or other UK country) and research status (research intensive or non-research intensive) were also recorded for the medical school. Research-intensive universities were defined as those that were members of the ‘Russell group’, which defines itself as ‘a group of leading UK universities who are committed to maintaining high quality research along with an exceptional learning and teaching experience’.

The data were extracted by JM with 10% of the images (selected at random) reviewed by a second researcher (ME or KM). Any differences of opinion were resolved through discussion, involving the third author where necessary.

### Comparator data

Comparator data were identified against which to compare the image bank. Demographic data were obtained about the UK public from the Office of National Statistics using the latest census [19]. National Health Service (NHS) Digital was used to collect demographic data for doctors and nurses [20]. The General Medical Council Medical School Annual Return was used to gather demographic data for medical students [21]. The Russell Group website was used to ascertain the Russell Group status for each medical school [22]. Demographic data for teachers were obtained from the Higher Education Statistics Agency [23]. Where there was not a specific comparator data group, UK census data were used [19].

### Data analysis

Descriptive statistics were calculated for each category. Chi-squared goodness of fit test was used to compare the image bank to the comparator data, since the data were categorical, and aimed to compare observed frequencies and expected frequencies. For comparison using the chi-squared goodness of fit, for ethnicity, the categories of other, mixed, and unknown were combined into one group. If the group had a value of 0, then a value of 1 was assigned. For the results of the chi-squared goodness of fit, the cut off for rejecting or accepting the null hypothesis was set at *p* < 0.05 for statistical significance. Statistical analysis was conducted using IBM SPSS 24. The database was also analyzed by different groupings of medical school: research intensive and non-research intensive; and by UK region: England and other UK countries.

### Hypotheses

Four null hypotheses guided the data analysis, as follows:

1. There will be no significant differences in the proportion of females and males in the images identified and the comparator data.
2. There will be no significant differences in how ethnicity is represented in the images identified and the comparator data.
3. The proportion of the overall themes of the images will be equal.
4. The proportions of the two groups of assumed specialties will be equal.

### Research ethics

Ethical approval was obtained from the MSc Clinical Education program team, operating with delegated authority on behalf of the University of Exeter Medical School’s Research Ethics Committee.

## Results

### Description of images

A total of 650 images, including 1,817 people, met the inclusion criteria. The median number of images per medical school was 19, with a median number of 2 people per image. Twenty medical schools were identified as being research intensive and 13 were not; 25 were identified as being in England and 8 in other UK countries.

Table 1 provides data relating to the assumed role of people in the images. Of the people in the images, 78% were assumed to be students (*n* = 1,423), 6% doctors (*n* = 108), 6% teachers (*n* = 100), 4% patients (*n* = 70), 2% nurses (*n* = 37) and 4% had ‘other roles’ (*n* = 79).

**Table 1:** Data on assumed roles and image information

Data group	Image	People	Student	Teacher	Doctor	Patient	Nurse	Other
Entire dataset (%)	650 (100)	1817 (100)	1423 (78)	100 (6)	108 (6)	70 (4)	37 (2)	79 (4)
Research intensive (%)	384 (59)	1083 (60)	879 (81)	60 (6)	57 (5)	35 (3)	18 (2)	34 (3)
Non-research intensive (%)	266 (41)	734 (40)	544 (74)	40 (5)	51 (7)	35 (5)	19 (3)	45 (6)
England (%)	511 (79)	1421 (78)	1110 (78)	74 (5)	90 (6)	52 (4)	30 (2)	65 (5)
Other UK countries (%)	139 (21)	396 (22)	313 (76)	26 (8)	18 (5)	18 (5)	7 (2)	14 (4)

Table 2 provides data relating to the setting of the images. Only 2% of the images (14/650) portrayed a community setting, whereas 24% (154/650) depicted a hospital setting and 40% of the images had another setting. Similarly, only 9% of the doctors (10/108) portrayed a community-based specialty, and 91% (98/108) portrayed a hospital-based specialty. These trends were consistent across groups (Tab. 2).

**Table 2:** Data on overall theme of the image and the assumed specialty group of the portrayed doctors

Data Group	Academic	Community	Hospital	Other	Community-based doctor	Hospital-based doctor
Entire dataset (%)	223 (34)	14 (2)	154 (24)	259 (40)	10 (9)	98 (91)
Research intensive (%)	142 (37)	6 (2)	81 (21)	155 (40)	6 (11)	51 (89)
Non-research intensive (%)	81 (30)	8 (3)	73 (27)	104 (39)	4 (8)	47 (92)
England (%)	179 (35)	13 (3)	117 (23)	202 (40)	9 (10)	81 (90)
Other UK countries (%)	44 (31)	1 (0)	37 (26)	57 (43)	1 (6)	17 (94)

Table 3 provides data relating to the ethnicity and gender of the people in the images. The largest ethnic group category portrayed was ‘White/White British’, accounting for 73% of people (*n* = 1,328), followed by 22% ‘Asian/Asian British’ (*n* = 401) and 5% ‘Black/Black British’ (*n* = 88). Whilst the percentages fluctuated slightly across the different assumed roles and groups of data the order stayed the same, apart from people who were assumed to be nurses, where there was a larger proportion of people who were assumed to be ‘Black/Black British’ (14%) than people who were assumed to be ‘Asian/Asian British’ (5%).

**Table 3:** Data for assumed gender and assumed ethnicity

Data group	Female	Male	Asian	Black	White	Other
Entire dataset (%)	1025 (56)	792 (44)	401 (22)	88 (5)	1328 (73)	0 (0)
Student (%)	823 (58)	600 (42)	364 (26)	74 (5)	985 (69)	0 (0)
Teacher (%)	36 (36)	64 (64)	13 (13)	2 (2)	85 (85)	0 (0)
Doctor (%)	41 (38)	67 (62)	15 (14)	4 (4)	89 (82)	0
Patient (%)	34 (49)	36 (51)	2 (3)	1 (1)	67 (96)	0 (0)
Nurse (%)	36 (97)	1 (3)	2 (5)	5 (14)	30 (81)	0 (0)

### Hypothesis 1—Assumed gender

The first null hypothesis was accepted for all but two groups of data (Tab. 4), i.e. the proportions of male to female gender for people in the images is approximately as you would expect based on national data amongst students, doctors, patients, and teachers. The proportions of male to female gender was significantly different to that expected in the images of nurses and ‘other’, although the numbers are small. Additionally, the null hypothesis was accepted for all the subgroups for the assumed role of students (i.e. the images appropriately represent the proportions of male to female gender amongst medical students).

**Table 4:** Results for chi squared goodness of fit

Data for	Observed Frequency	Expected frequency	Chi squared	Degree of freedom	*P* value
Sex	Entire dataset	Census	1.045	1	0.307
	Research intensive	Census	1.276	1	0.259
	Non-research intensive	Census	0.676	1	0.411
	England	Census	1.000	1	0.317
	Other UK countries	Census	2.632	1	0.105
	Doctor	NHS	2.151	1	0.143
	Other roles	Census	14.612	1	<0.001
	Patient	Census	0.143	1	0.705
	Nurse	NHS	6.839	1	0.009
	Teacher	HESA	3.792	1	0.052
	Student	GMC	0.514	1	0.473
	Student—research intensive	GMC	0.842	1	0.359
	Student—non-research intensive	GMC	0.099	1	0.753
	Student—England	GMC	0.362	1	0.547
	Student—other UK countries	GMC	1.251	1	0.263
Ethnicity	Entire dataset	Census	37.343	3	<0.001
	Research intensive	Census	36.052	3	<0.001
	Non-research intensive	Census	39.314	3	<0.001
	England	Census	24.676	3	<0.001
	Other UK countries	Census	51.949	3	<0.001
	Doctor	NHS	35.646	3	<0.001
	Other roles	Census	1.510	3	0.680
	Patient	Census	5.636	3	0.131
	Nurse	NHS	14.031	3	0.003
	Teacher	HESA	12.910	3	0.005
	Student	GMC	12.378	3	0.006
	Student—research intensive	GMC	12.364	3	0.006
	Student—non-research intensive	GMC	14.921	3	0.002
	Student—England	GMC	13.161	3	0.004
	Student—other UK countries	GMC	11.886	3	0.008
Theme	Entire dataset	Equal	33.440	3	<0.001
	Research intensive	Equal	36.560	3	<0.001
	Non-research intensive	Equal	28.636	3	<0.001
	England	Equal	32.188	3	<0.001
	Other UK countries	Equal	37.099	3	<0.001
Speciality	Entire dataset	Equal	67.240	1	<0.001
	Research intensive	Equal	70.560	1	<0.001
	Non-research intensive	Equal	60.840	1	<0.001
	England	Equal	64.000	1	
	Other UK countries	Equal	72.516	1	<0.001

*NHS* National Health Service Digital, *HESA* Higher Education Statistics Agency, *GMC* General Medical Council

### Hypothesis 2—Assumed ethnicity

The second null hypothesis was rejected for students, doctors, teachers, nurses, but accepted for patients and ‘other’ (Tab. 4). The proportions of the ethnicity of people represented in the images were not as expected for all healthcare professionals and students. Further analysis by medical school subgroups suggested that no group of medical schools was accurately representing the proportions expected for ethnicity.

### Hypothesis 3—Assumed setting

The third null hypothesis was rejected across the board. Community settings were significantly underrepresented in the images portrayed, compared with hospital settings and/or academic settings (Tab. 4).

### Hypothesis 4—Assumed specialty group

The fourth null hypothesis was also rejected across the board (Tab. 4). Community specialists were significantly underrepresented in the images portrayed, compared with hospital specialists (Tab. 4). Of the 108 people who were assumed to be a doctor, only 10 of them were assumed to have a community-based specialty. Statistics from NHS Digital regarding full-time equivalents for general practitioners (GPs) and hospital consultants, show GPs make up 41.95%, and hospital consultants make up 58.05% of senior doctors, meaning the percentages shown in this research of 9.26% for community-based doctors and 90.74% for hospital doctors fall significantly short of the comparator data [24].

## Discussion

This research set out to answer the question ‘To what extent do the images of people used within UK medical school prospectuses and websites reflect current demographic data for medical students and healthcare professionals?’. A large dataset of 650 images portraying 1,817 people was compiled and analyzed against comparator data. This research adds value to the existing literature by evaluating the impact of images medical schools use, and how these might be affecting personal contact factors identified by Foster [5] and the intangible factors identified by Brown [6]. It also builds upon research done by Chowdhury et al. into the types of learning environments displayed by medical schools [9].

Our analysis showed the proportion of female to male people in the images was appropriately represented, alleviating concern about potential barriers to women joining the medical profession. While the factors involved with making such decisions are complex and poorly understood, it is imperative that gender is accurately represented in the images used to attract prospective students so as to avoid any potential discrimination or creation of barriers.

However, the proportions of different ethnicities were substantially different to that which would be expected based on the comparator data for medical students, doctors, nurses, and teachers. This implies that generally ethnicity is poorly reflected by the images used. Applying image theory, the photos have a significant potential to be negatively influencing peoples’ decision making by violating fundamental values around diversity and equality. Framing this finding in the context of social identity theory, the images used could be posing a barrier for people from ethnic minority groups from applying to medical school, as the images do not show it to be as ethnically diverse as would be expected. Considering the impact images have on decision making and perceptions, having official images showing a less ethnically diverse group of people than the comparator data shows, undermines widening access to medicine. It is possible that potential medical students who would make excellent doctors are put off before they even apply for a place at medical school.

Given the challenges in recruiting to general practice in the UK, the underrepresentation of community settings and specialties in the images we analyzed is surprising. The key role of community placements in medical education appears to be significantly underplayed in the images chosen, which may serve to undermine the recent initiatives by regulatory bodies and universities. Research by Chowdhury et al. analyzed the use of images in prospectuses for the depiction of different learning environments; they found similarly low proportions of UK medical schools representing community placements as a place of learning, with only 2 of the 32 prospectuses depicting community placements [9]. This shows the underrepresentation of community placements is not a new phenomenon but unfortunately has not changed in the last 9 years. This builds on the signals medical schools are sending with the overall theme of the image, and image theory suggests that this longstanding preference for hospital-based images may be putting off potential students from choosing general practice as their specialty.

It is interesting to reflect upon why there was under-representation in the images used on medical school websites. Although our research was not designed to capture these reasons, we suspect they result from subtle unconscious bias when selecting images, perhaps based on nothing more sinister than judgements about what will make for a ‘good’ or ‘interesting’ or ‘exciting’ photograph. Images portraying fast moving and high technology hospital placements may be thought to be more visually appealing for a photograph than community placements, for example. However, the impact of these judgements is far-reaching and we suggest they should be made by those with in depth knowledge of the relative proportions outlined in this research.

### Strengths and Limitations

A strength of this study is the large dataset collected and analyzed, comprising 650 images and 1,817 people. Another strength is that the data analysis was hypothesis-led, leading to a more rigorous approach to interpreting the results. A limitation of the research is that most data extraction was undertaken by a single researcher, with only 10% verified by a second researcher, and thus prone to subtle biases and influences. Similarly, the data that could be collected, for example on ethnicity, were relatively crude. Another limitation of this study is that the number of webpages and images which were excluded was not recorded due to the limited resources available for the project. As this study only included images from UK medical schools it is important to be cautious about generalizing the findings to other country settings, further research internationally would be valuable to ascertain if these findings are generalizable.

## Implications for policy and practice

It is important that medical schools update the images displayed on their websites and in their prospectuses, to accurately portray the diverse applicants they aspire to recruit and to portray the range of career choices available to these applicants through the images they choose.

We suggest that medical schools worldwide examine and evaluate their websites, prospectuses, and other promotional material for implicit messages they may be sending, which may support or undermine the rhetoric of widening access to medicine and the subsequent messages about the medical workforce they would like to create.

To ensure that there are sufficient drivers and accountability for medical schools to enact this change, key stakeholders such as regulatory or licencing bodies, should be involved to ensure the issues highlighted are dealt with moving forward.

To build on this study, qualitative research with potential applicants to medical school would be helpful to ascertain the extent these images have on influencing applicants’ choices. Additionally, alignment of promotional images could be evaluated by monitoring for any changes in applicants and by reviewing current student career intentions and graduate destinations.

Whilst this research has focussed on UK medical schools, the importance and benefits of a multicultural and diverse medical workforce are not limited to the UK. Thus, we suggest that the findings and recommendations of this research are likely to be transferrable to other country settings, although future research beyond the UK with similar study designs will be needed to confirm this.

## Conclusion

In conclusion, this research has found that, while the images used by medical schools are representative of the expected proportions for gender, they are not representative of ethnicity, and this should be addressed. Additionally, the number of images which show community placements or community-based doctors is much lower than those of hospital placements or hospital-based doctors. By altering the proportion of community themed images, it may be possible to attract prospective students for whom this represents the career path they want. At the very least it sets the expectation early that a substantial proportion of medical students will become GPs, so the medical workforce can meet the challenges of the future. Medical schools should consider signalling theory, image theory and social identity theory as well as their potent impacts on students’ decision making when selecting images to display on their websites or prospectuses.

## References

[1] Department of Health (2018). Expansion of undergraduate medical education—government response to consultation 2017.

[2] Roberts N, Bolton P (2018). Medical school places in england from September (2018)—briefing paper: the house of common library.

[3] Health Education England (2018). New medical schools to open to train doctors of the future: Health Education England.

[4] Amin M, Chande S, Park S, Rosenthal J, Jones M (2018). Do primary care placements influence career choice: what is the evidence?. Educ Prim Care.

[5] Foster K (2014). Medical school choice: what influences applicants?. Clin Teach.

[6] Brown C (2007). A qualitative study of medical school choice in the UK. Med Teach.

[7] Cleland JA, Johnston PW, Anthony M, Khan N, Scott NW (2014). A survey of factors influencing career preference in new-entrant and exiting medical students from four UK medical schools. Bmc Med Educ.

[8] McHarg J, Mattick K, Knight LV (2007). Why people apply to medical school: implications for widening participation activities. Med Educ.

[9] Chowdhury S, Chowdhury R, Sandars J (2009). A picture is worth a thousand words: the use of visual imagery in medical school application prospectuses in the UK. Med Teach.

[10] Alexander K, Fahey PT, Nicholson S, Cleland J (2017). ‘Why not you?’ Discourses of widening access on UK medical school websites. Med Educ.

[11] Visual Imagery RJ (1982). Applications to advertising. Adv Consum Res.

[12] Pike S (2002). Destination image analysis—a review of 142 papers from 1973 to 2000. Tour Manag.

[13] Spence M (1973). Job market signaling. Quart J Econ.

[14] Beach LR (1990). Image theory: decision making in personal and organizational contexts.

[15] Beach LR (1993). Image theory: an alternative to normative decision theory. Adv Consum Res.

[16] Tajfel H (1974). Social identity and intergroup behaviour. Soc Sci Inform.

[17] Haggarty L (1996). What is content analysis?. Med Teach.

[18] Medical Schools Council (2017). Medical schools: Medical Schools Counci.

[19] Office for National Statistics (2011). UK census.

[20] NHS Digital (2017). Staff by staff group, grade, gender and ethnicity.

[21] General Medical Council (2016). Medical school annual return 2016–17.

[22] Russell Group (2018). Russell group website.

[23] Higher Education Statistics Agency (2018). Data and analysis—staff.

[24] NHS Digital (2018). Statistics show change in NHS workforce over time.

